# 190. Outcomes of Early Ceftaroline-based Combination Therapy for Methicillin-resistant *Staphylococcus aureus* Bacteremia

**DOI:** 10.1093/ofid/ofab466.392

**Published:** 2021-12-04

**Authors:** Mackenzie Dolan, Megan Shah, James A Platts-Mills, Zachary Elliott, Gregory Madden, Joshua Eby

**Affiliations:** 1 University of Virginia Health, Charlottesville, Virginia; 2 University of Virginia, Charlottesville, VA; 3 Division of Infectious Diseases & International Health, Charlottesville, VA; 4 University of Virginia Health System, Charlottesville, Virginia

## Abstract

**Background:**

Monotherapy with vancomycin (VAN) or daptomycin (DAP) remains the guideline-driven standard of care for methicillin-resistant *Staphylococcus aureus* bacteremia (MRSA-B) despite concerns regarding efficacy. While combination therapy is often utilized as salvage treatment for persistent MRSA-B, growing data suggest a potential benefit of combination therapy with ceftaroline as initial therapy for MRSA-B. In light of these data, we updated practice guidance at our institution for management of MRSA-B in March 2020 to favor initial combination therapy with ceftaroline. Herein, we present an assessment of outcomes of patients with MRSA-B initiated on early combination therapy.

**Methods:**

This was a single-center, retrospective, cohort study of adult patients admitted to the University of Virginia with MRSA-B between July 1, 2018 and February 28, 2021. Patients were considered to have received combination therapy if they received VAN or DAP plus ceftaroline (CPT) within 5 days of index blood culture, and monotherapy if during that period they received VAN and/or DAP alone. The primary outcome was a composite of persistent bacteremia, 30-day all-cause mortality, and 30-day bacteremia recurrence. Time to microbiological cure and safety outcomes were also assessed. A propensity score-weighted logistic regression was conducted. A post-hoc analysis of the primary composite outcome was performed in which patients were only deemed to have received combination therapy if it was started within 72 hours.

**Results:**

Of 94 patients included, 57 received monotherapy (55 VAN, 2 DAP) and 37 received combination therapy with CPT (30 VAN, 7 DAP). There was no difference between groups for the primary composite outcome in the primary analysis (OR 2.7, 95% CI 0.95-7.72) or the post-hoc analysis (OR 2.37, 95% CI 0.68-8.22). Time to microbiological cure was not different between groups (mean difference 1.47, 95% CI 0.20-2.74). Safety outcomes were similar.

**Conclusion:**

In this retrospective study, there was no clear benefit or harm of early initiation of combination therapy for MRSA-B. Additional study of initial combination therapy with ceftaroline is warranted given the small number of subjects in the study presented.

**Disclosures:**

**All Authors**: No reported disclosures

